# Intensive Training Reveals Plasticity in the Adult Auditory System

**DOI:** 10.1371/journal.pbio.0040104

**Published:** 2006-03-07

**Authors:** Liza Gross

When your exuberant kitten races too far up a tree to negotiate a safe descent, you can quickly locate your distressed charge by homing in on her plaintive cries. In the wild, mammals from Norway rats to elephant seals rely on sound localization skills to escape threats, forage, and communicate. The brain networks underlying these skills are largely shaped by experience during development. Though the adult brain retains some capacity for rewiring these networks, it's not clear which tasks benefit from plasticity or how the brain engenders this flexibility.

In a new study, Oliver Kacelnik, Andrew King, and colleagues investigated these questions by manipulating the hearing of adult ferrets. They show that ferrets with obstructed hearing (an earplug in the left ear) can quickly adapt to altered auditory cues when trained to do so in the service of a meaningful task (getting a drink). Frequent training was crucial in boosting the rate and extent of their improvement.

To localize sound, the brain processes spatial cues in the sound waves that enter each ear. In interpreting sounds on the horizontal plane (imagine a coyote approaching from the side), the brain uses disparities in how sounds reach each ear, called interaural time differences and interaural level differences. Changes in level at different frequencies (the sound's spectral properties) are produced by the external ear and can reveal the source's elevation (think swooping raptor), whether it's coming from front or back, and sometimes help with monaural localization.

To study adaptive hearing in an adult mammal, the authors blocked the left ears of ferrets with specially outfitted earplugs and measured their ability to localize sounds in the horizontal plane. To do this, the authors trained ferrets to approach a sound source to get a water reward. The ferrets licked a waterspout, triggering a burst of noise from one of twelve loudspeakers. Ferrets that approached the correct speaker received the reward. Performance was based on accuracy—did they approach the sound source?—and on head-orienting responses (which were compared with unconditioned, pre-plug head-orienting behavior).

Ferrets were presented with sound bursts lasting either 1,000 or 40 milliseconds. Without earplugs, ferrets had no trouble localizing the 1,000-millisecond sounds; they were slightly less adept at localizing the brief bursts. With earplugs, performance dropped considerably for both bursts. Ferrets had the most trouble with sounds coming from their left (obstructed) side, but errors increased significantly for all 12 sound sources.

The authors retested one group of earplugged animals every six days to see if they could use the altered cues to recover their localization skills. By three weeks, the trained animals had shown a marked improvement, localizing sounds coming from the right (unobstructed) side about as well as they had before the ear was plugged. The speed and accuracy with which ferrets recovered their localization abilities depended on training, with more complete recovery occurring when the animals received more frequent training.

There is some evidence to suggest that visual cues might help to boost auditory plasticity. To test this possibility, the authors compared the earplugs' behavioral effects on two groups of ferrets: one blind since infancy, and another with normal vision. Both groups could localize about the same without earplugs, made more errors after receiving the plug, and then steadily improved with the periodic retesting. These results show that the ferrets could not only be trained to reprocess abnormal localization cues but also that they could do so without visual cues. Then, by training another group of ferrets to localize both auditory and visual stimuli before inserting the earplug, the authors show that sound localization depends “exclusively on auditory training” and does not involve “a visual recalibration of auditory space.” Furthermore, adaptation did not depend on error feedback, since rewards were not based on performance.

After removal of the earplugs, ferrets made errors reflecting a small bias toward the previously plugged ear: they initially processed the cues as if the ear was still plugged. This aftereffect, though transient, indicates that the adaptive response relies in part on reinterpreting the relationship between binaural cues and location. However, compensation for the earplug-disrupted binaural cues mainly involved the animals' learning to make use of other cues that were less distorted by the earplug, including low-frequency interaural time differences, and spectral cues provided by the unobstructed ear.

Altogether, these results show that the adult auditory system can adapt to abnormal spatial cues and can do so rapidly with intensive training. By recording brain activity as animals perform the tasks described here, future studies can shed light on the brain regions responsible for this plasticity. Whatever the mechanism, the finding that plasticity follows targeted, intensive training suggests that patients with hearing disorders might benefit from a similar strategy—providing more evidence that an old brain can sometimes learn new tricks.

## 

**Figure:**
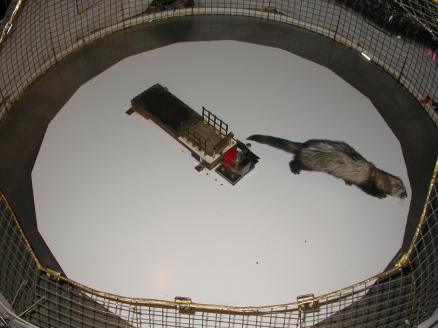
Measuring the accuracy with which ferrets can localize sound.

